# Azoles resistance of candida species causing vulvo-vaginitis in reproductive age women at a tertiary care setting

**DOI:** 10.12669/pjms.38.8.5984

**Published:** 2022

**Authors:** Ronaq Zaman, Ihsan Ullah, Humera Adeeb, Ambreen Arif

**Affiliations:** 1 Dr. Ronaq Zaman, MBBS, MPhil. Assistant Professor of Pathology, Department, Kabir Medical College, Peshawar, Pakistan; 2 Dr. Ihsan Ullah, MBBS, DFM, PGD EBM, HPE, PhD. Associate Professor, Institute of Pathology & Diagnostic Medicine, Khyber Medical University, Peshawar, Pakistan; 3 Dr. Humera Adeeb, MBBS, MPhil. MHPE, FCPS-II Trainee, Community Medicine Department, Khyber Medical College, Peshawar, Pakistan; 4 Ambreen Arif, MPhil. Pakistan Health Research Council, Research Centre Khyber Medical College, Peshawar, Pakistan

**Keywords:** Broth micro-dilution, Disc diffusion, High vaginal swabs, Prevalence, Reproductive age, Vulvovaginal candidiasis

## Abstract

**Objectives:**

To study prevalence and resistance pattern to azoles of candida species causing vulvovaginitis in reproductive age women.

**Methods:**

Samples were collected from Hayatabad Medical Complex from November 2018 to May 2019. This cross-sectional study was conducted at the department of Microbiology, Institute of Pathology and Diagnostic Medicine, Khyber Medical University Peshawar, Pakistan. A total of 369 high vaginal swabs were collected. Candida was isolated by vaginal swabs inoculation on Sabouraud’s dextrose agar (SDA). Colonies on SDA were inoculated on Candida CHROM agar to identify candida species. Wet film microscopy and Gram staining were performed. Biochemical identification was done with 20C AUX. Antifungal susceptibility testing was done by disc diffusion and broth micro-dilution methods to find the resistance pattern of azole drugs. Fluconazole, Clotrimazole, Miconazole, Voriconazole and Itraconazole were the azoles drugs used.

**Results:**

Among 43%(n=158) positive candida cases, 44%(n=85) were non pregnant women while 41%(n=73) were pregnant. The Candida species distribution of 158 isolates was as follow; *Candida albicans* *(C. albicans)* 46.2%(n=73), *Candida krusei* *(C. krusei)* 29.1%(n=46), *Candida parapsilosis (C. parapsilosis)* 19%(n=30) and *Candida glabrata* *(C. glabrata)* 5.7%(n=9). Overall Candida isolates were highly resistant 72%(n=113) to Fluconazole while least resistant 21.5 % (n=34) to Itraconazole.

**Conclusion:**

*C. albicans* is the most prevalent specie involved in Vulvovaginal candidiasis. Candida species were found to be least resistant to Itraconazole followed by Voriconazole, Miconazole, Clotrimazole and Fluconazole.

## INTRODUCTION

Candida is the most important opportunistic pathogen among yeast. The Candida spectrum of infection is wide, ranging from local mucosal involvement to life-threatening disseminated infections. Vulvovaginal candidiasis (VVC) is the most common infection of female lower genital tract caused by Candida species. It has been reported that 75% of women get at least one episode of vulvovaginal candidiasis during their lives and 50% of them have multiple episodes.^1^

*Candida albicans* is the most prevalent specie causing vulvovaginitis. There is a rise in infection rate due to non-albican species like *C. glabrata, C. krusei, C. parapsilosis, C. tropicalis*, *C. dublinensis* and some rare species. Non-albican species are involved in recurrent candidiasis and are more resistant to antifungal drugs. *Candida krusei* is inherently resistant to Fluconazole while *C. glabrata* is only 3-7% of resistant.^2^

Vulvovaginal candidiasis is more common in the reproductive age and pregnancy due to increase in estrogen level, which causes deposition of glycogen that favors growth of Candida. Risk factors are antibiotic use, oral contraceptives, intrauterine contraceptive device, pregnancy, immunocompromised conditions like steroids use, Diabetes Mellitus, and chemotherapy.^3^

In the past few decades, there is improvement in the antimicrobial testing and focus remained on cost effective, precise and reproducible testing methods. Clinical Laboratory Standards Institute (CLSI) guidelines are used as reference scheme available for yeast susceptibility testing, where disc diffusion and broth micro dilution methods are considered for antifungal susceptibility testing.^4^

Topical and oral azoles are conventionally used to treat vulvovaginal candidiasis. The most common anti-fungal used to treat vulvovaginal candidiasis is Fluconazole due to its easy availability and oral dosage Clotrimazole and Miconazole are the commonly used topical azoles in our set up. There is a rise in Candida strains resistant to one or more of the azoles due to their widespread use. The newer Azoles like Voriconazole and Itraconazole have broad spectrum activity against the Fluconazole resistant Candida species. Cross resistance has been reported in Fluconazole resistant Candida species against the newer Azoles.^5^

In our clinical set up, bacterial culture and sensitivity of the specimens are commonly done but fungal cultures are not done. There is no national surveillance program to monitor the prevalence of Candida species and antifungal resistance. Only direct microscopy of the specimens is performed to report Candida species while identification of the Candida at species level and antifungal susceptibility testing are not done. Keeping this present state of dealing with Candida, this project was designed to study the prevalence of different Candida species and their resistance pattern.

## METHODS

This cross-sectional study was conducted at the Microbiology department, Institute of Pathology and Diagnostic Medicine, Khyber Medical University Peshawar, Pakistan. After approval of the project by the advanced studies research board of Khyber Medical University Peshawar, ethical approval was obtained from the Institutional ethical committee, with reference number. DIR/KMU AS & RB/IM/00081 dated 30-October-2018. Samples were collected from Hayatabad Medical Complex from November 2018 to May 2019. The sample size was calculated by taking relevant references from literature using OpenEpi calculator at confidence level 95% and bound of error 5%.^6^ Sampling technique was nonprobability convenient sampling. All women of childbearing age (married) both pregnant and nonpregnant having signs and symptoms of vulvovaginitis were included in the study. Women taking antifungal drugs were excluded. Written informed consent was obtained from all patients. After enrolment in the study, demographic data were collected from patients using a structured questionnaire and two high vaginal swabs were collected from each patient using aseptic techniques.^7^ Samples were then inoculated on Sabouraud’s dextrose agar. Candida present in the samples were grown as creamy white colour colonies which were then sub-cultured on Candida CHROMagar for species identification.^8^

Wet film microscopy and Gram staining were also performed for identification. The isolates were identified by carbohydrate assimilation method using API 20C AUX system (Bio Merieux). Pure Candida isolates were stored at -80 °C in micro bank.^9^

Susceptibility testing to Azoles was conducted according to the Clinical and Laboratory Standards Institute guidelines. For quality control American Type Culture Collection (ATCC) 90028 was used. Fluconazole (25μg), Clotrimazole (10 μg), Miconazole (50μg), Voriconazole (1μg) and Itraconazole (10μg) (Himedia) were tested by using disc diffusion method (DD). Candida colonies were transferred to saline. Turbidity was matched with 0.5 McFarland standards. The suspension was inoculated on Muller Hinton agar with the help of a swab. Filter paper discs containing antifungals were applied on the Muller Hinton media. Interpretation of antifungal was done according to the Clinical and Laboratory Standards Institute guidelines - M44A document.

Broth micro-dilution (BMD) was performed by micro-titer plate method following M27A CLSI guidelines. Inoculum was prepared by picking Candida colonies from 24 hours culture on Sabouraud’s dextrose agar. The colonies were suspended in 5ml of 0.85% saline and adjusted to 0.5 McFarland standards. The suspension was diluted 1:50 followed by a dilution of 1:20 in Roswell Park Memorial Institute Medium RPMI 1640 (M1972 HI media part A, D glucose was added). Stock solutions of Clotrimazole, Miconazole, Itraconazole and Voriconazole (Sigma, USA) were prepared in dimethyl sulfoxide (DMSO) at concentration of 1600 μg/ml. Fluconazole was prepared in distilled water in concentration of 1280μg/ml. Final concentration of Fluconazole (64-0.125 μg/ml) and Clotrimazole, Miconazole, Itraconazole and Voriconazole (16- 0.03μg/ml) were made in RMPI 1640 media. Drug dilutions inoculum of 100μl was transferred to the column 1-10 of the 96-well plates (Nest, China). Column 11 and 12 were growth control and sterility control, respectively. Inoculum of 100μl was added to columns 1-11. Reading of MIC were taken after 24-48 hours. Minimum inhibitory concentration (MIC) break-points for Fluconazole, Itraconazole and Voriconazole were determined according to M27A document.^10^ For Miconazole and Clotrimazole, there are no interpretive breakpoints available in CLSI document. For Clotrimazole resistant breakpoint of ≥1μg/ml reported was used.^11^ For Miconazole resistant breakpoint of ≥16μg/ml was used.^12^

### Statistical Analysis:

All data were transferred to excel sheet and analyzed by using SPSS version 20. Frequencies and percentages were calculated for different categorical variables. Mean and standard deviation was calculated for numerical variables like age and zone of inhibition. Percent agreement was calculated for disc diffusion and broth micro-dilution methods. Data was presented in the form of tables.

## RESULTS

A total of 369 patients were included in the study. High vaginal swabs were collected from all patients where Candida species were isolated from 158(43%) cases. Out of total 369, 193 patients were non-pregnant women with 85 (44%) positive vaginal swabs while 176 pregnant with 73(41%) positive for vulvo-vaginal candidiasis. Among pregnant patients, 13(17.81%) were in the first trimester, 40(54.79%) were in the second trimester, and 20(27.40%) were in the third trimester.

Age distribution among 369 patients was as follows; 66(18%) were in the range of 18-23 years, 121(33%) were in the range of 24-29 years, 105(28%) were in the range of 30-35 years, 53 (14%) were in range of 36-41 years, 24(7%) were in range of 42-46 years. Mean age and standard deviation were 30 years ±6.37 respectively.

Age distribution among positive 43%(n=158) patients was as follows; 30(19%) were in the range of 18-23 years, 51(32%) were in the range of 24-29 years, 49(30%) were in the range of 30-35 years, 23 (15%) were in range of 36-41 years, 5(3%) were in range of 42-46 years. Mean age and standard deviation were 29.512 years and ±6.08 respectively.

Age distribution among 44%(n=85) of nonpregnant patients was as follows; 13 (15.2%) were in age range of 18-23 years, 28 (33%) were in range of 24-29 years, 26(30%) were in the range of 30-35 years. Fifteen (17.6%) were in range of 36-41 years. 3(3.5%) of the patients were in range of 42-46 years. Mean age and standard deviation for non-pregnant women were 30 years and ± 6.15 respectively.

Age distribution among 73 pregnant patients was as follows; 15 (21%) of patients were in age range of 18-23 years, 27 (38%) of patients were in age range of 24-29 years, 24(33%) of the patients were in the range of 30-35 years, 6 (8%) of patients were in range of 36-41 years, one (1.3%) of the patients were in range of 42-46 years. Mean age and standard deviation for pregnant women were 28.78 years and ± 5.95 respectively.

Candida species distribution found among 158 of isolates was as follow; *Candida* *albicans* 73(46.2%), *Candida krusei* were 46 (29.1%), *Candida parapsilosis* were 30 (19%) and *Candida glabrata* were nine isolates (5.7%).

By disc diffusion method candida species showed susceptibility pattern as follows; Fluconazole was resistant in 75.9%, susceptible dose dependent in 1.9% and sensitive in 22.2% of isolates. Clotrimazole was sensitive in 20.3%, SDD in 15.2% and resistant in 64.6% of isolates. Miconazole was sensitive in 18.4%, SDD in 16.5 and resistant in 65.2% of isolates. Voriconazole was sensitive in 36.7, SDD in 2.5% and resistant in 60.8% of isolates. Itraconazole was sensitive in 64.6%, SDD 11.4% in and resistant in 24.1% of isolates as shown in Table-I.

**Table-I:** Percent susceptibility pattern of Candida species to Azoles by disc diffusion method.

Candida Species	Fluconazole			Clotrimazole			Miconazole			Voriconazole			Itraconazole		

	S	R	SDD	S	R	SDD	S	R	SDD	S	R	SDD	S	R	SDD
C. albicans	38.4	57.5	4.1	34.2	57.5	8.2	28.8	54.8	6.4	47.9	50.7	1.4	65.8	30.1	4.1
C. krusei	0	100	0	8.7	67.4	23.9	8.7	71.7	19.6	30.4	65.2	4.3	73.9	6.5	19.6
C. parapsilos	16.7	83.3	0	6.7	73.3	20	10	76.7	13.3	20	80	0	36.7	46.7	16.7
C. glabrata	22.2	77.8	0	11.1	77.8	11.1	11.1	77.8	11	33.3	55.6	11.1	66.7	22.2	11.1
Total	22.1	75.9	1.8	0.3	64.6	15.2	18.4	65.2	16.5	36.7	60.8	2.5	64.6	24.1	11.4

*S = Sensitive, *R = Resistant, *SDD = Susceptible dose dependent.

Mean of zones of inhibition of Fluconazole, Clotrimazole, Miconazole, Voriconazole and Itraconazole were 12.97mm,13.26mm ,13.09mm 14.90mm and 24.92mm and standard deviation were ± 6.39, ±6.36, ±6.30, ± 7.77 and ±10.85 respectively.

By broth microdilution method candida species showed susceptibility pattern as follows; Fluconazole, was sensitive in 39 (24.7%), SDD in 5 (3.2%) and resistant in 114 (72.2%) of isolates. Clotrimazole was sensitive in 60 (38%) and resistant in 98 (62.0%) of isolates. Miconazole was sensitive in 63 (39.9%) and resistant in 95 (60.1) of isolates. voriconazole was sensitive in 68 (43%), SDD in 5 (3.2%) and resistant in 85 (53.8%) of isolates. Itraconazole was sensitive in 108(68.4), SDD in 14 (8.9%) and resistant in 36 (22.8%) of isolates as shown in Table-II. The non-albican Candida species showed higher resistance as compared to *Candida albicans* .Table-II.

**Table-II:** Percent susceptibility pattern of Candida species to Azoles by Broth Micro-dilution.

Candida Species	Fluconazole			Clotrimazole		Miconazole		Voriconazole			Itraconazole		

	S	R	SDD	S	R	S	R	S	R	SDD	S	R	SDD
C. albicans	46.6	49.3	4.1	39.7	54.8	46.6	53.4	49.3	47.9	2.7	65.8	30.1	4.1
C. krusei	0	100	0	13	65.2	32.6	67.4	34.8	60.9	4.3	82.6	4.3	13.0
C. parapsilos	13.3	80.0	0	13.3	70.0	33.3	66.7	36.7	63.3	0	53.3	33.3	13.3
C. glabrata	11.1	88.9	0	11.1	77.8	44.4	55.6	55.6	33.3	11.1	66.7	2.2.2	111.1
Total	24.7	72.2	3.2	38	62.0	39.9	60.1	43.0	53.8	3.2	68.4	22.8	8.9

*S = Sensitive, *R = Resistant, *SDD = Susceptible dose dependent.

Of the resistant *Candida albicans* isolates 36 had MIC of 64μg/ml (fluconazole), 40 had MIC in range of 1-16μg/ml (clotrimazole), 39 had MIC of 16μg/ml (miconazole), 35 had MIC in range of 4-16μg/ml (voriconazole), 22 had MIC in range of 1-16μg/ml (itraconazole). Table-III.

**Table-III:** Azoles MIC^BMD^ (μg/ml) distribution among Candida isolates n= (%).

MIC μg/ml	0.03	0.06	0.12	0.25	.5	1	2	4	8	16	32	64
***C. albicans***												
Fluconazole			1(1.3)	1(1.3)	3(4.1)	11(15)	3(4.1)	6(8.2)	9(12.3)	1(1.3)	2(2.8)	36(49)
Clotrimazole		2(1.3)	11(15)	7(9.5)	13(18)	9(12.3)	5(9)	12(16)	8(10)	6(8.2)		
Miconazole		3(4.1)		1(1.3)	4(5.4)	3(4.1)	6(8.2)	5(9)	14(19)	39(53)		
Voriconazole	1(1.3)	3(4.1)	7(9.5)	4(5.4)	8(10)	12(16)	2(2.8)	12(16)	9(12)	14(19)		
Itraconazole	1(1.3)	20	27	2(2.8)	1(1.3)	2(2.8)	3(4.1)	4(5.4)	7(9.5)v	6(8.2)		
***C. krusei***												
Fluconazole												46(100)
Clotrimazole				5(11)	11(23)	5(11)	7(15)	6(13)	5(11)	7(15)		
Miconazole				3(6.5)		2(4.3)	11(23)	48.6)	5(10.8)	31(67)		
Voriconazole			1(2.1)	4(8.6)	5(11)	6(13)	2(4.3)	8( )	11(23)	9(19)		
Itraconazole	4(8.6)	15(32)	19(41)	3(6.5)	5(11)		1(2.1)		1(2.1)			
***C. parapsilosis***												
Fluconazole					1(3.3)	1(3.3)		1(3.3)	1(3.3)		2(6.6)	24(80)
Clotrimazole			2(6.6)	2(6.6)	5(16)	2(6.6)	5(16)	8(26)		6(20)		
Miconazole					2(6.6)	1(3.3)	3(10)		4(13)	20(66)		
Voriconazole		6(20)	10(33)	2(6.6)	2(6.6)	1(3.3)	3(10)	2(6.6)	3(10)	1(3.3)		
Itraconazole	2(6.6)	4(13)	10(33)	4(13)		5(16)	2(6.6)		1(3.3)	2(6.6)		
***C. Glabrata***												
Fluconazole						1(11)						8(88)
Clotrimazole				1(11)	1(11)	1(11)	1(11)		3(33)	2(22)		
Miconazole						1(11)	1(11)		2(22)	5(55)		
Voriconazole				1(11)	1(11)	3(33)	1(11)		2(22)	1(11)		
Itraconazole		2(22)	4(44)	1(11)		2(22)						

A total of 46 resistant *Candida krusei* isolates had MIC of 64μg/ml (fluconazole), 30 had MIC in range of 1-16 μg/ml (clotrimazole), 31 had MIC of 16μg/ml (miconazole), 28 had MIC in range of 4-16μg/ml (voriconazole), 2 isolates had MIC in range of 1-8μg/ml (itraconazole) Table-III.

A total of 24 resistant *candida parapsilosis* isolates had MIC of 64μg/ml (fluconazole), 20 had MIC in range of 1-16 μg/ml (clotrimazole), 20 had MIC of 16μg/ml (miconazole), 5 had MIC in range of 4-16μg/ml (voriconazole), 10 had MIC in range of 1-16μg/ml (itraconazole) as shown in Table-III.

Of the resistant *Candida glabrata* isolates eight had MIC of 64μg/ml (fluconazole), seven had MIC in range of 1-16μg/ml (clotrimazole), five had MIC of 16μg/ml (miconazole), five had MIC in range of 4-16μg/ml (voriconazole), and two isolates had MIC of 1μg/ml (itraconazole) as shown in Table-III.

There was a good agreement between DD and BMD methods in identifying the percentage of resistant Candida species for Fluconazole, Clotrimazole, Miconazole, Voriconazole and Itraconazole. However, some isolates showed resistance to azoles antifungals by DD method were found to be sensitive by BMD method. Percent agreement between the two antifungal susceptibility methods for Fluconazole, Clotrimazole, Miconazole, Voriconazole and Itraconazole was found to be 94% ,82%, 95%, 90% and 86% respectively.

## DISCUSSION

Among opportunistic fungi candida species are the most important. Vulvovaginal candidiasis is one of the common infections in females of reproductive age. It is estimated that 75% of child bearing age females have at least one episode of VVC during their lifetime.^13^ Numerous studies have been performed to establish the prevalence of vulvovaginal candidiasis. The prevalence found in the present study is compatible to other studies for example 40% prevalence of VVC is reported in Pakistan.^14^ Prevalence rates of 45% and 44.8% are reported in Uganda and Lebanon respectively, which are similar to the current study.^7^,^15^ Higher rate (48.4%) of Vulvovaginal candidiasis is reported in India.^16^ Lower infection rate i.e., 28.3%, and 22.71% are reported by other researchers.^17^,^18^ This may be related to cultural, geographical, climatic and socioeconomic conditions.

In the present study, the age range of 18-46 years was observed, which is in agreement to studies conducted by various researchers who reported higher incidence in the age group of 15-45 years. This may be due to the high levels of estrogen during reproductive age and use of contraceptives.^19^ Our findings are not in agreement with the findings of a study conducted in Nigeria which reported a high incidence of *candida albicans* during 36-40 years of age while low prevalence (20.42%) in 20-25 years age group.^20^

The current study revealed high prevalence of VVC among second trimester of pregnancy of 54.79% followed by 27.40% in third trimester. Study published in Nepal reported 61% in second trimester.^21^ Prevalence of 54% in 2nd trimester, 30% in 3^rd^ trimester and 16% in 1^st^ trimester was reported in India.^22^ These studies are in agreement to the current study.

A study done in Brazil, it was found that *candida albicans* is the most prevalent specie Causing VVC, While *C.glabrata* is reported second common specie in that study which is not similar to present study which found *C.krusei* as the second common specie.^23^

The results of susceptibility pattern regarding Itraconazole are similar to a study done in Kenya, which revealed Itraconazole was sensitive in 88.33% of isolates. This is in agreement with our study while resistance to Fluconazole and Clotrimazole found is lower than our study.^24^ Resistance to Fluconazole, Voriconazole and Itraconazole was in agreement with those found in a study in Turkey.^5^ Candida species were most sensitive to voriconazole in another study done in Pakistan study while in our study isolates were more sensitive to Itraconazole drug.^4^ In a study conducted in China Candida species revealed infrequent resistance.^25^ In another study conducted in Iran results showed 8 (25.8 %) and 6 (7.8 %) of resistance in *C. glabrata and C. albican* isolates to Fluconazole, respectively. Furthermore, resistance to Voriconazole and Itraconazole were observed in 8.4%, and 3.7% of stains.^26^ The resistance rates of Fluconazole, Clotrimazole and Miconazole of the current study are higher than other population because of the irrational and excessive use of these drugs. A good agreement between DD method and BMD was reported in India which is similar to that found in this study.^27^

### Limitations:

This study was done only in one hospital of Peshawar. It might increase our knowledge about the causative species and susceptibility pattern of azoles antifungal drugs if samples are collected from different hospitals.

## CONCLUSION

This study showed that *C.albicans* is the main causative agent followed by *C.krusei, C.parapsilosis and C.glabrata*. Antifungal sensitivity testing of Candida species against azoles showed higher sensitivity to Itraconazole followed by Voriconazole, Miconazole, Clotrimazole and Fluconazole. Therefore, Itraconazole is a good therapeutic choice in vulvovaginal candidiasis management. A good agreement was found between DD and BMD methods of susceptibility testing. Disc diffusion method is less laborious and is more convenient to perform.

### Authors Contribution:

**RZ:** Did Literature search, data collection, laboratory work and manuscript Writing.

**IU:** Concept & project design, review, manuscript writing, editing and final approval of manuscript.

**HA:** Data analysis and drafting of manuscript.

**AA:** Did data analysis and interpretation.
